# Breaking the 30-day barrier: Long-term effectiveness of a nurse-led 7-step transitional intervention program in heart failure

**DOI:** 10.1371/journal.pone.0279815

**Published:** 2023-02-07

**Authors:** Lidia Alcoberro, Pedro Moliner, Joan Vime, Santiago Jiménez-Marrero, Alberto Garay, Sergi Yun, Alexandra Pons-Riverola, Raúl Ramos-Polo, Mar Ras-Jiménez, Marta Tajes, Encarna Hidalgo, Esther Calero, Marta Ruiz, Nuria José-Bazán, Carles Ferre, Cristina Delso, Laia Alcober, Cristina Enjuanes, Josep Comin-Colet

**Affiliations:** 1 Bellvitge Biomedical Research Institute (IDIBELL), Bio-Heart Cardiovascular Diseases Research Group, L’Hospitalet de Llobregat, Barcelona, Spain; 2 Department of Cardiology, Bellvitge University Hospital, L’Hospitalet de Llobregat, Barcelona, Spain; 3 Department of Cardiology, Community Heart Failure Unit, Bellvitge University Hospital, L’Hospitalet de Llobregat, Barcelona, Spain; 4 Department of Clinical Sciences, School of Medicine, University of Barcelona, Barcelona, Spain; 5 Department of Internal Medicine, Bellvitge University Hospital, L’Hospitalet de Llobregat, Barcelona, Spain; 6 Emergency Department Short-Stay Unit, Bellvitge University Hospital, L’Hospitalet de Llobregat, Barcelona, Spain; 7 Institut Catala de la Salut, SAP Delta Primary Care Service, El Prat de Llobregat, Barcelona, Spain; Karolinska Institutet, SWEDEN

## Abstract

**Background and aims:**

Heart failure (HF) programs successfully reduce 30-day readmissions. However, conflicting data exist about its sustained effects afterwards and its impact on mortality. We evaluated whether the impact of a new nurse-led coordinated transitional HF program extends to longer periods of time, including 90 and 180 days after discharge.

**Methods and results:**

We designed a natural experiment to undertake a pragmatical evaluation of the implementation of the program. We compared outcomes between patients discharged with HF as primary diagnosis in Period #1 (pre-program; Jan 2017—Aug 2017) and those discharged during Period #2 (HF program; Sept 2017—Jan 2019). Primary endpoint was the composite of all-cause death or all-cause hospitalization 90 and 180 days after discharge. 440 patients were enrolled: 123 in Period #1 and 317 in Period #2. Mean age was 75±9 years. There were more females in Period #2 (p = 0.025), with no other significant differences between periods. The primary endpoint was significantly reduced in the HF program group, at 90 [adjusted OR 0.31 (0.18–0.53), p <0.001] and at 180 days [adjusted OR 0.18 (CI 0.11–0.32), p <0.001]. Such a decrease was due to a reduction in cardiovascular (CV) and HF hospitalization. All-cause death was reduced when a double check discharge planning was implanted compared to usual care [0 (0%) vs. 7 (3.8%), p = 0.022].

**Conclusion:**

A new nurse-led coordinated transitional bundle of interventions model reduces the composite endpoint of all-cause death and all-cause hospitalization both at 90 and 180 days after a discharge for HF, also in high-risk populations. Such a decrease is driven by a reduction of CV and HF hospitalization. Reduction of all-cause mortality was also observed when the full model including a more exhaustive discharge planning process was implemented.

## Introduction

Heart failure (HF) is a devastating syndrome with a highly negative impact on mortality, morbidity, and health-related quality of life of patients with this diagnosis [1–3]. HF also represents a challenge to healthcare systems due to its growing prevalence and to the rising medical resource use and expenditure associated to the syndrome [4–6]. Those rates are particularly high in vulnerable populations, such as the elderly and those with multiple comorbidities [7,8].

Suboptimal organization of care and poor patient self-care have been identified as factors that may partially explain the occurrence of major adverse events and limitations in patient-reported outcomes in the setting of HF [9,10]. Regarding this, multidisciplinary HF disease management programs have been established to improve results through a structured follow-up based on patient education, optimization of medical treatment and improved access to care. Evidence has shown that HF programs improve quality of life for HF patients and successfully reduce 30-day readmission and its associated healthcare costs [11–13].

However, conflicting data have been reported by other studies about the sustained beneficial effects of multidisciplinary HF disease management programs beyond 30 days and its impact on mortality [14–17]. Moreover, as readmission and mortality are competing risks, in many cases the counterpart of reducing 30-day readmission has been a mortality increase. Consequently, recent recommendations suggest extending the period of evaluation of such programs to 90 and 180 days after discharge [18].

Given the limitations of previous studies above mentioned, our study sought to assess the results of the implementation of a new nurse-led coordinated transitional bundle of interventions model in our integrated Health Care Area and evaluate its long-term impact on the occurrence of major adverse HF events (MAHFE) including all-cause mortality, cardiovascular mortality, HF hospitalization and the composite endpoint of all-cause death or all-cause hospitalization in a broad-spectrum cohort of real-world patients with chronic HF. Importantly, we evaluated the influence of this multidisciplinary HF program, initially directed to reduce 30-day readmission and mortality, on longer periods of time, including 90 days and 180 days after discharge.

## Methods

### Health care setting

The setting of this intervention is the South Metropolitan Barcelona integrated health care area. In this urban area, care for HF is provided either in 10 Primary Care centres or in a university hospital with comprehensive Cardiology, Internal Medicine and Emergency Departments, allowing the care and specialized treatment of the full spectrum of cardiac conditions. Data from all patients attending our hospital are systematically codified and collected in a database that contains exhaustive demographical and clinical variables such as the patient’s codified ICD-9 and ICD-10 diagnosis, pharmacological treatment, laboratory information, emergency room consultations, hospital admissions and vital status. It also includes the Adjusted Morbidity Group (AMG) of the patient, a method for grouping morbidity adapted to the Spanish healthcare System that identifies target populations with multimorbidity and higher use of healthcare resources [19]. Individual patients are assigned a code that allows dissociation from personal data and further data extraction for anonymized data analysis.

Within the framework of the creation of specialized units for the care of chronic conditions such as HF [20], in 2017 the hospital-based community Heart Failure Unit (HFU), as the hospital component of the South Metropolitan Barcelona comprehensive, integrated, primary care-hospital community HF program, was established following an integrated care model previously developed by our group in a different health care setting [11,21]. Our aim was to develop a multidisciplinary program which amalgamates the care processes and services in primary and hospital care for HF patients through a hospital based multidisciplinary HFU that comprises Cardiologists, Internists, General Practitioners, and specialized nurses trained in HF, including innovations in the process of nurse led-led interventions such as bundles of structured interventions. The care innovations introduced in this project allowed the definition of a new 7-step nurse-led day-care hospital-based transitional bundle of interventions care model. This differentiated pathway was embedded in a new model of integrated transitional care for HF developed in the South Metropolitan Barcelona Area (South Metropolitan Barcelona model) and initiated in the University Hospital Bellvitge and Delta del Llobregat Primary Care Service (Catalan Institute of Health).

Since the conception of the program, all patients admitted for HF undergo a psycho-social and clinical comprehensive in-hospital evaluation as a part of the nurse-led day-care hospital based 7-step transitional intervention, shown in **S1 Table**. These evaluation and associated interventions seek to establish an individualized follow-up plan in the most suiting healthcare environment for each patient profile. Patients who have preserved functionality that allows them to leave their house with minimal assistance or support and attend hospital outpatient visits and also have features of high risk of readmissions such as ventricular dysfunction [i.e., left ventricular ejection fraction (LVEF) <50%] or ≥2 HF-related admissions in the last six months regardless of LVEF, are deemed candidates to be enrolled in the nurse-led day-care hospital-based multidisciplinary pathway originally directed to decrease 30-day readmissions and 30-day mortality. Although the program coordinates all patients with the diagnosis of HF, for the present analysis we focused on these patients at high risk of readmission that were candidates to be enrolled in the nurse-led structured follow up conducted in our outpatient HF day-care hospital. High-risk frail patient unable to attend outpatient visits, along with those already on home-based care and patients with intermediate-low risk of readmission are excluded from this outpatient hospital-based intervention. The subset of high-risk frail patients receives home-based care by case managers at home using a dedicated clinical pathway. Patients with intermediate-low risk receive primary care-based care by primary care doctors, nurses and primary care cardiologists using a dedicated clinical pathway.

The 7-step nurse-led day-care hospital-based transitional bundle of interventions care pathway consists of a 3 to 6 months holistic and intensive intervention that alternates face-to-face visits and telematic (telephone and videoconference) contacts with the aim of educating and empowering patients on self-care, prescribing pharmacological and non-pharmacological treatments based on Clinical Practice Guidelines (CPGs) recommendations [22], delving into the etiological study of HF when indicated and identifying and treating associated comorbidities and HF decompensations. HFpEF patients are followed for 3 months prioritizing educational aspects, while patients with HFrEF complete a 6-month intervention to allow treatment up-titration. The follow-up period can be extended if the prespecified program goals are not attained. The process of intervention consists in several different interventions in multiple dimensions (bundles) and is supported by well-structured clinical pathways with checklists and other support decision tools to allow nurses managing interventions with autonomy (guided care) and having physician back-up when needed.

### Study design

We designed a natural experiment in our health care area that included all consecutive patients that were discharged alive after hospitalization with HF as primary diagnosis between January 2017 and January 2019 fulfilling eligibility criteria. Main inclusion criteria were: 1) hospital discharge with HF as primary diagnosis, 2) preserved functionality that allows attending hospital outpatients visits, 3) LVEF <50% or ≥2 HF-related admissions in the last six months regardless of LVEF. Exclusion criteria were: 1) inability to attend outpatient visits, 2) home-based care patients, 3) LVEF ≥50% and <2 HF-related admissions in the last six months.

Data was retrospectively analysed from the administrative databases selecting patients fulfilling eligibility criteria and linking these patients with individual codes that allow dissociation from personal data and anonymized data analysis. From this database, information about clinically related readmissions and survival up to six months after discharge from the index hospitalization was obtained at individual level. The study was approved by the local committee of ethics for clinical research (Ref. PR339/21) and was conducted in accordance with the principles of the Declaration of Helsinki. Given the retrospective nature of the data obtained from administrative databases the need for consent was waived by the ethics committee.

For the purpose of this analysis, we compared the outcomes of patients discharged alive from our hospital with a primary diagnosis of HF in the period before the implementation of the HF program (January-2017-August-2017, period #1, control group) with patients discharged during Period #2 (Sept-2017-Jan-2019, HF program group), after the implementation of the 7-step bundle of interventions model. Patients of period #2 were divided in two additional periods: period #2.1 from Sept-2017 to April-2018 (single check in step #2 of the bundle of interventions) and period #2.2 from May-2018 to January 2019 (double check in step #2of the bundle of interventions), as shown in **S1 Fig**. Retrospective follow-up was 6 months for all groups.

### Allocation of patients into control and intervention groups

Therefore, patients in the HF program group were enrolled in the new nurse-led day-care hospital based 7-step transitional intervention while patients in the control group (usual care group) would have fulfilled inclusion criteria in the new hospital-based nurse-led pathway (HFU) but were discharged in the period preceding the implementation of the program. A careful and thorough retrospective review of the administrative databases was carried out to ensure that eligibility criteria were met in the control group patients. Due to that circumstance our study is a natural experiment, that is, an observational study in which the exposure to the experimental (i.e., the HF program) and control conditions is determined by the temporal period in which patients were discharged after hospitalization for HF.

A description of the data sources and the coding criteria for the study are provided in **S2 Table**. For both the diagnosis of HF and clinically related admissions, we used the criteria recommended in the Chronic Condition Indicator of the Agency for Healthcare Research and Quality [23]. For the index admission and successive clinically related readmissions, we considered only unplanned acute admissions of more than 12 hours’ duration, following the same criteria used in administrative analyses conducted previously by our group [1,5,6,21].

### Study endpoints

The primary endpoint was a composite of all-cause death or all-cause hospitalization 30, 90 and 180 days after discharge from the index hospitalization. Secondary endpoint variables were the individual components of the primary endpoint, the number of cardiovascular (CV) hospitalizations, HF hospitalizations, a composite of all-cause death and CV hospitalization and a composite of all-cause death and HF hospitalization.

### Subgroup analysis

Subgroup analyses were performed to evaluate the effect of a multidisciplinary HF program in particular populations with high risk of adverse outcomes, such as the elderly and those with chronic kidney disease (CKD) or considered as complex chronic patients (CCP) by their Primary Care physician. We defined as elderly the patients aged ≥ 80 years. Patients were classified as having CKD according to the thresholds of eGFR (in ml/min/1.73m2) proposed by the National Kidney Foundation as non-CKD (eGRF≥60), CKD (eGRF<60), stage III CKD (eGRF 60–30), stage IV (eGFR 30–15) and stage V (eGFR 15–0). The CCP is a label used in the Primary Care setting in Catalonia for patients with a particular profile of comorbidity, chronicity and person-specific socioeconomic, cultural or environmental dimensions that interfere with the delivery of usual care and require the implementation of specific individual plans [24].

As additional analysis we explored the relative outcome of conducting the discharge plan using a single (period 2.1) or double check (period 2.2) approach as described in **S1 Table.**

### Statistical analysis

All patients included in the HF program were analysed for the study, hence we did not perform a priori power calculation. Demographic and clinical characteristics, as well as laboratory test results, were summarized using basic descriptive statistics, both overall and categorized by the pre-specified periods and vulnerable subgroups. For quantitative variables, arithmetic mean and standard deviation or median and interquartile ranges were reported, and p values were obtained using the t-test. For qualitative variables, number and percentages within specified groups were calculated using the χ2 test for the p values. Logarithmic transformation was used to fit skewed continuous variables, such as NT-proBNP, into normal distributions when necessary. All multivariable models were adjusted by age, sex, LVEF, aetiology, BMI, eGFR, Hb and NT-proBNP levels.

Univariate and multivariable binary logistic regression models (forward stepwise) were performed with several demographic and HF severity related parameters to predict outcomes, which fitted a normal distribution. To confirm the results, we performed a time to first analysis using multivariable adjusted cox proportional hazard models. The missing data were handled by omitting the missing values.

Finally, to explore possible differences in HF program effect in high-risk populations, we repeated the different analyses based on whether age was ≥80 years or <80 years, eGFR was <60 mL/min or ≥60 mL/min and whether they were catalogued as CCP by their primary care physician or not (non-CCP). All p values reported were constructed with a type-I error alpha level of 5% with no adjustments for multiplicity and p values <0.05 were considered statistically significant. Data were managed with the SPSS statistical package, version 25 (IBM SPSS, New York, USA) and R software (version 4.0.4; R Foundation for Statistical Computing, Vienna, Austria).

## Results

### Baseline characteristics

From January 2017 through January 2019, a total of 440 patients were included in the study (N = 440): 123 in the pre-program period (Period #1, January 2017 to August 2017) and 317 in the HF program period (Period #2, September 2017 to January 2019). The amount of missing data was very scarce, as we only had missing data in 64 (14.5%) NT-proBNP levels values.

The baseline characteristics of the study population, both overall and according to treatment group, are shown in **Table 1**. Mean age of the whole cohort was 75 ± 9 years. There was a higher proportion of female patients in Period #2 (38.2% vs. 26.8%, p-value = 0.025), with no differences in other baseline characteristics, including age, LVEF, morbidity index, eGFR or NT-proBNP status (all p-values >0.05). The mean LVEF was 46.2 ± 16% and 43.9% of patients had an ejection fraction of 50% or greater (HFpEF). Burden of comorbidities was high among the study population, with 66.8% of patients being diagnosed with CKD, 67.5% atrial fibrillation and 79.3% AMG ≥4 (i.e., high or very high morbidity).

**Table 1:** Demographics and baseline characteristics of the overall study population and according to intervention group.

Variables	Total (n = 440)	Usual Care (n = 123)	HF Program (n = 317)	p-value
Age, years	75.4 ± 9	75.2 ± 10	75.5 ± 9	0.755
Sex (female), No. (%)	154 (35.0)	33 (26.8)	121 (38.2)	0.025
BMI, Kg/m^2^	28.2 ± 5.0	28.2 ± 4.7	28.2 ± 5.1	0.965
LVEF, No. (%)	46.2 ± 15.7	47.1 ± 15.6	45.9 ± 15.8	0.461
HFrEF, No. (%)	173 (39.3)	50 (40.6)	123 (38.8)	0.768
HFmrEF, No. (%)	74 (16.8)	14 (11,4)	60 (18.9)	0.067
HFpEF, No. (%)	193 (43.9)	59 (48.0)	134 (42.3)	0.280
Ischemic cause of HF, No. (%)	159 (36.1)	50 (40.7)	109 (34.4)	0.220
**Comorbidities, No. (%)**				
Hypertension	382 (86.8)	107 (87.0)	275 (86.8)	0.947
Atrial Fibrillation	297 (67.5)	83 (67.5)	214 (67.5)	0.995
Dyslipidaemia	305 (69.3)	85 (69.1)	220 (69.4)	0.952
Diabetes Mellitus	204 (46.4)	57 (46.3)	147 (46.4)	0.995
Chronic Kidney Disease	294 (66.8)	82 (66.7)	212 (66.9)	0.966
Chronic Obstructive Pulmonary Disease	141 (32.1)	45 (36.7)	96 (30.3)	0.204
Anaemia Status‡	39 (8.9)	13 (10.6)	26 (8.2)	0.433
**Group Stratification (Clinical Complexity)**				
Adjusted Morbidity Group (AMG)≥4, No. (%)	349 (79.3)	98 (79.7)	251 (79.2)	0.908
**Laboratory measurements**				
eGFR-ml/min/1.73m^2^	50.3 ± 21.8	50.8 ± 23.9	50.1 ± 21.0	0.756
NT-proBNP>1000 pg/mL, n(%)	284 (75.5)	75 (81.5)	209 (73.6)	0.124

Anaemia status was defined as moderate to severe anaemia (Hb levels≤100g/dl).

BMI denotes body mass index, eGFR estimated glomerular filtration rate, HF heart failure, HFpEF heart failure with preserved ejection fraction, LVEF left ventricular ejection fraction, NT-proBNP N-terminal pro–B-type natriuretic peptide.

### Primary endpoints

During the first 30 days after discharge the primary composite endpoint was significantly reduced in the HF program period: it occurred in 21 (17.1%) patients in Period #1 and in 22 (6.9%) patients in Period #2 (crude odds ratio [OR] 0.36 [95% CI 0.19–0.69]; adjusted OR 0.29 [95% CI 0.14–0.60]; p-value = 0.002).

The primary composite outcome of all-cause hospitalization or all-cause mortality at 90 days occurred in 40 patients (32.5%) in Period #1 and in 48 (15.1%) patients in Period #2 (crude OR 0.37 [95% CI 0.23–0.60]; adjusted OR 0.31 [95% CI 0.18–0.53]; p-value <0.001). At 180 days the primary composite endpoint occurred in 61 patients (49.6%) in Period #1 and in 71 (22.4%) patients in Period #2 (crude OR 0.27 [95% CI 0.18–0.43]; adjusted OR 0.18 [95% CI 0.11–0.32]; p-value <0.001) (**Table 2**).

**Table 2:** Multivariable adjusted binary logistic regression analyses evaluating the outcome of HF program management vs. usual care on clinical outcomes 30, 90 and 180 days after discharge.

	TIME POINTS					
	30 DAYS		90 DAYS		180 DAYS	
**Primary Endpoint**	*Odds Ratio (95% CI)	p-value	*Odds Ratio (95% CI)	p-value	*Odds Ratio (95% CI)	p-value
All cause death or all cause hospitalization	0.29 (0.14–0.60)	0.001	0.31 (0.18–0.53)	<0.001	0.18 (0.11–0.32)	<0.001
**Secondary Endpoints**						
HF hospitalization	0.15 (0.06–0.36)	<0.001	0.14 (0.08–0.27)	<0.001	0.13 (0.07–0.22)	<0.001
CV hospitalization	0.17 (0.07–0.41)	<0.001	0.16 (0.09–0.30)	<0.001	0.13 (0.07–0.22)	<0.001
All-cause hospitalization	0.27 (0.13–0.57)	<0.001	0.29 (0.17–0.51)	<0.001	0.19 (0.11–0.33)	<0.001
All-cause death	- ^	- ^	0.81 (0.14–4.65)	0.813	0.37 (0.11–1.32)	0.126
All-cause death or CV hospitalization	0.20 (0.09–0.46)	<0.001	0.19 (0.10–0.34)	<0.001	0.13 (0.07–0.23)	<0.001
All cause death of HF hospitalization	0.17 (0.07–0.40)	<0.001	0.15 (0.08–0.28)	<0.001	0.13 (0.08–0.23)	<0.001

*Comparison of HF Program vs. Usual Care (reference category).

×The multivariable model was adjusted for: Age, sex, LVEF, aetiology, BMI, eGFR, Hb and NT-proBNP levels.

CI denotes confidence interval, CV cardiovascular, HF heart failure.

^ There were no deaths during the first 30 days.

**Fig 1:**
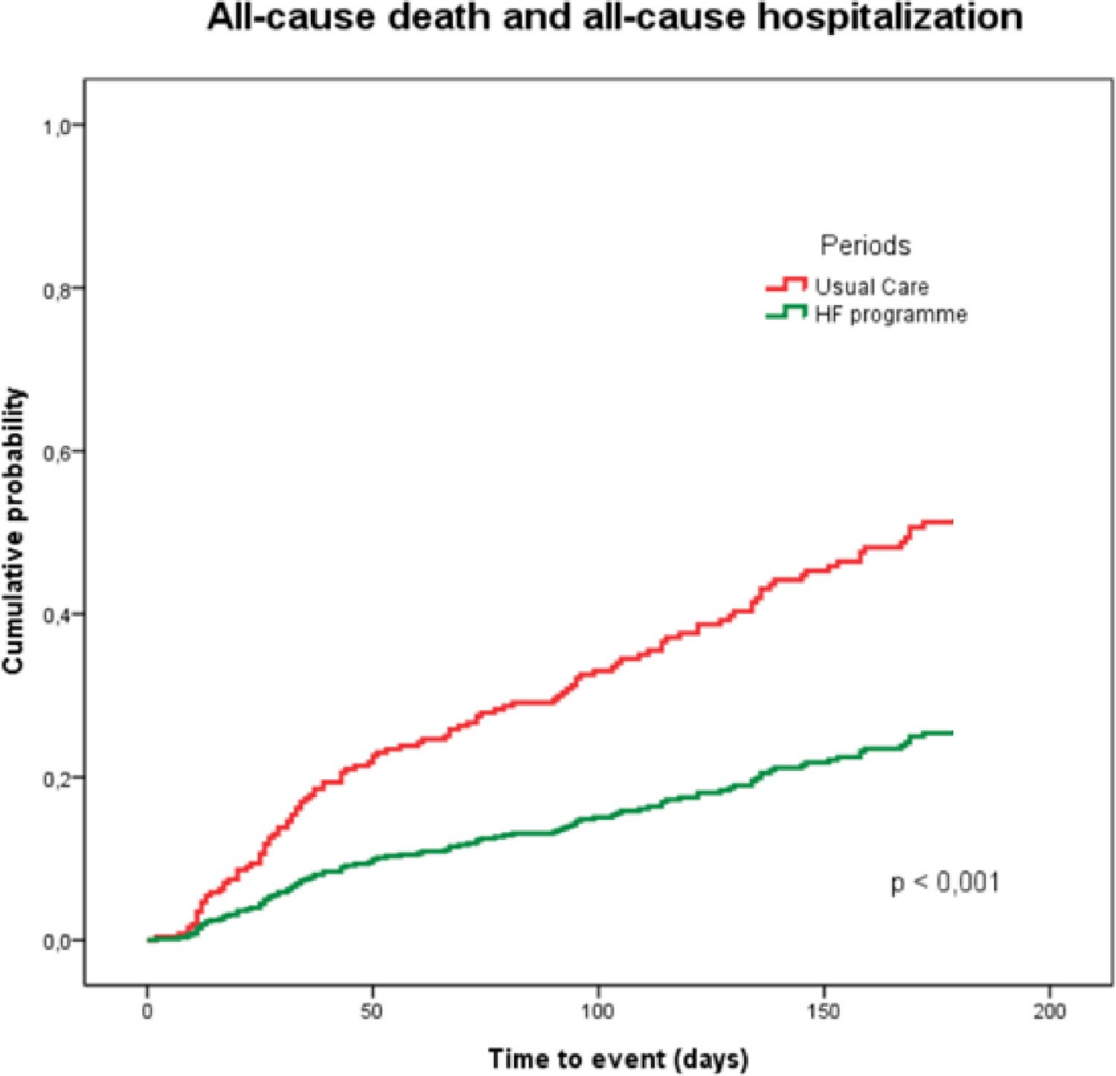
Adjusted time-to-event estimates for the primary endpoint according to treatment group.

In prespecified covariate-adjusted Cox model analyses, the estimated hazard ratio in Period #2 as compared with Period #1 was 0.40 ([95% CI 0.27–0.57]; p-value <0.001) (**Table 3 and Fig 1**). The results of the HF program were consistent across both sex strata: HR 0.40 (95% CI 0.26–0.63) for men and HR 0.40 (95% CI 0.20–0.83) for women.

**Table 3:** Multivariable adjusted Cox regression analyses evaluating the outcome of HF program management vs. usual care on clinical outcomes.

	Usual Care (n = 123)			HF Program (n = 317)				
**Primary Endpoint**	Total Events	Patients with Event	Event Proportion (%)	Total Events	Patients with Event	Event Proportion (%)	*Hazard ratio (95% CI)	p-value
All cause death or all cause hospitalization	114	62	50.4	120	76	24.0	0.40 (0.27–0.57)	<0.001
**Secondary Endpoints**								
HF hospitalization	81	50	40.7	53	37	11.7	0.22 (0.14–0.34)	<0.001
CV hospitalization	86	54	43.9	62	45	14.2	0.25 (0.16–0.38)	<0.001
All-cause hospitalization	108	60	48.7	113	72	35.6	0.39 (0.27–0.57)	<0.001
All-cause death	6	6	4.9	7	7	2.2	0.61 (0.19–1.97)	0.411
All-cause death or CV hospitalization	92	57	46.3	69	51	16.1	0.26 (0.17–0.39)	<0.001
All cause death of HF hospitalization	87	53	43.1	60	43	13.6	0.24 (0.15–0.37)	<0.001

*Comparison of HF Program vs. Usual Care (reference category).

×The multivariable model was adjusted for: Age, sex, LVEF, aetiology, BMI, eGFR, Hb and NT-proBNP levels.

CI denotes confidence interval, CV cardiovascular, HF heart failure.

### Secondary endpoints

**Fig 2:**
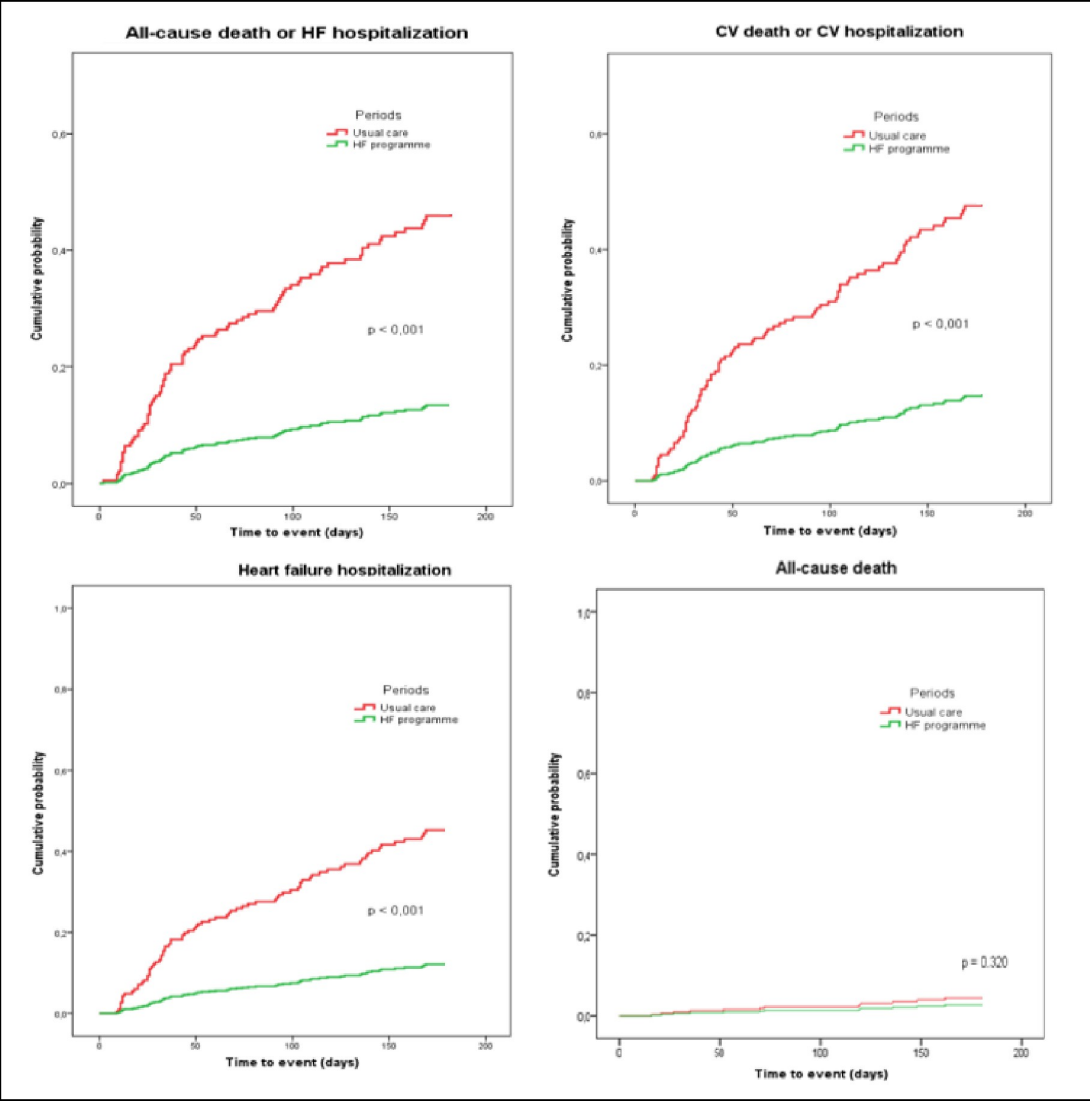
Adjusted time-to-event estimates for the secondary endpoints according to treatment group.

Event rates for the all-cause hospitalization component of the primary composite endpoint favoured the HF program. In Period #1, 60 patients (48.8%) were hospitalized at 180 days, as compared with 67 patients (21.1%) in Period #2 (adjusted OR 0.19 [95% CI 0.07–0.22]; p-value <0.001). Such a decrease was at expense of a reduction in CV hospitalization (43.9% versus 12.6%; adjusted OR 0.13; [95% CI 0.07–0.22]; p-value <0.001) and HF hospitalization (40.7% versus 10.4%; adjusted OR 0.13 [95% CI 0.07–0.22]; p-value <0.001) (**Fig 2**).

The other component of the primary composite outcome, all-cause death, was not significantly reduced in Period #2. All-cause death at 180 days occurred in 5 patients (4.1%) in Period #1 and in 7 patients (2.2%) in Period #2 (adjusted OR 0.19; [95% CI 0.03–1.06]; p-value 0.058).

The incidence of the secondary composite endpoint of all-cause death or HF hospitalization at 180 days was higher in Period #1 than in Period #2 (adjusted OR 0.13; [95% CI 0.08–0.23]; p-value <0.001). Similar results were obtained in the composite endpoint of all-cause death or CV hospitalization at 180 days (adjusted OR 0.13; [95% CI 0.07–0.23]; p-value <0.001).

### Special subgroups

**Fig 3:**
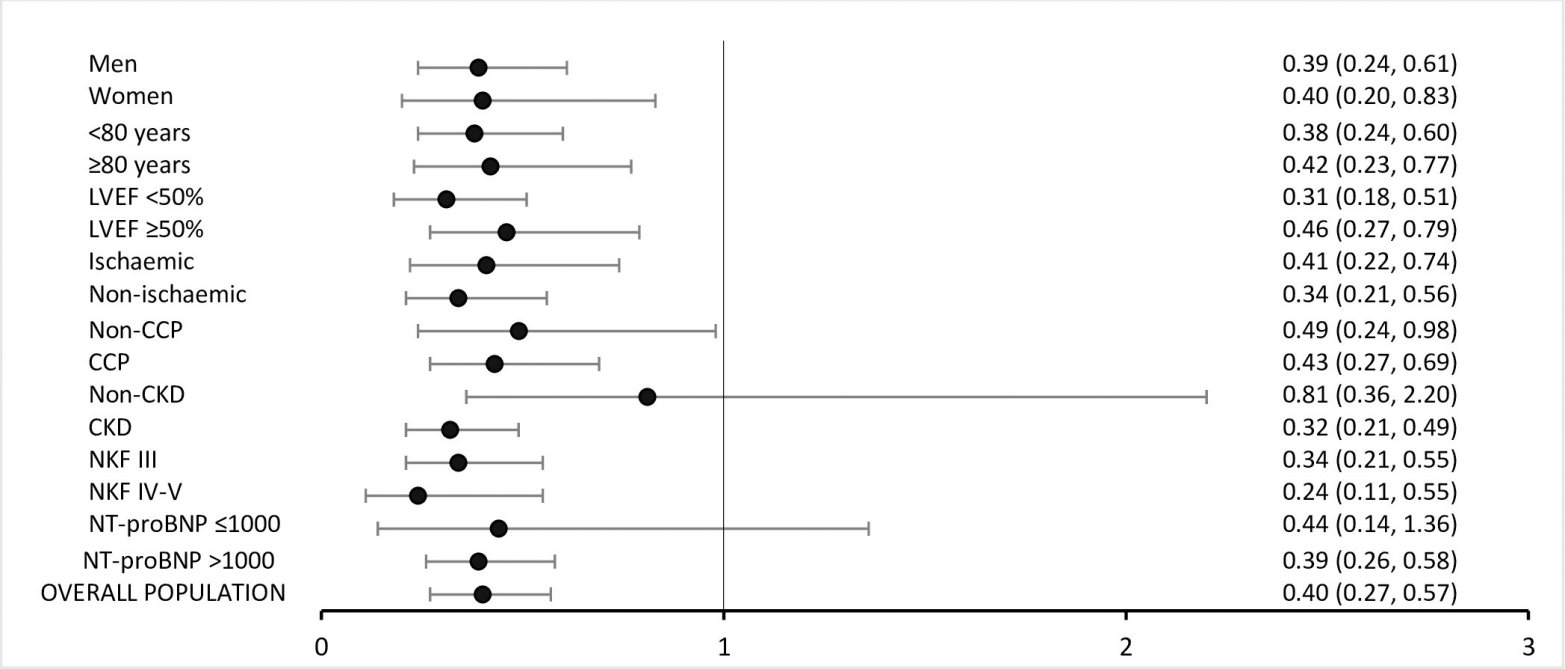
Multivariable binary logistic regression evaluating the outcome of HF program management vs. usual care on clinical outcomes in special subgroups.

As shown in **Fig 3**, similar results regarding the primary endpoint were observed across most prespecified vulnerable subgroups. The lower event rate in the HF program group was also maintained irrespective of other demographical and clinical variables such as sex, LVEF or HF aetiology.

The HF program results were similar in young and elderly patients. A lower risk of the primary endpoint was observed in the subgroup of patients aged ≥80 years old in Period #2 (adjusted HR 0.42; [95% CI 0.23–0.77]; p-value = 0.005) (**S3 Table and S2 Fig**).

Equally, CCP patients managed in our nurse-led integrated care-based HF program, compared to usual care, obtained similar results than non-complex patients: CCP patients managed in the HF program achieve a significant reduction of the primary composite endpoint (adjusted HR 0.41; [95% CI 0.26–0.66]; p-value <0.001) (**S3 Table and S2 Fig**) compared to the usual care group.

Moreover, in the subgroup of patients with CKD, the primary endpoint of all-cause mortality or all-cause hospitalization at 180 days was significantly reduced in Period #2 (adjusted HR 0.32; [95% CI 0.21–0.49]; p-value < 0.001). Among CKD patients, those with advanced CKD (defined as stage IV or V of CKD according to the NKF thresholds) also presented a significant reduction of the primary composite endpoint in Period #2 (adjusted HR 0.24; [95% CI 0.11–0.55]; p-value <0.001) (**S3 Table and S2 Fig)** compared to Period #1 before the implementation of the 7-step nurse-led bundle of interventions model.

Finally, we analysed the relative impact of conducting the discharge plan using a single check (Period #2.1, 182 patients) or double check (Period #2.2, 135 patients) approach (**S4 Table**). First, we compared the effects of these two different approaches with the usual care approach in terms of the primary endpoint. As shown in **S3 Fig**, either single check (adjusted HR 0.594, 95% CI [0.411–0.858]; p-value = 0.006) or double check approach (adjusted HR 0.311, 95% CI [0.192–0.505]; p-value<0.001) reduced the risk of all-cause death or clinically related readmission (all-cause) compared to the usual care approach. Moreover, as shown in **S4 Table** and **S4 Fig**, a more intensive discharge planning modality (double check approach) was associated with diminished risk of the primary end-point all-cause death or clinically-related readmission at 180 days (HR 0.53, 95% CI [0.32–0.860]; p-value = 0.011) compared to a single check approach, Interestingly, all-cause death was reduced in period #2.2 when a double check discharge planning was implanted compared to usual care (0 [0%] vs. 7 [3.8%] respectively, p-value = 0.022 for Fisher’s exact test) but also compared with single check approach (0 [0%] vs. 6 [4.9%] respectively, p-value **= 0.011 for Fisher’s exact test).**

## Discussion

In this pragmatic evaluation the implementation of an innovative 7-step nurse-led day-care hospital-based transitional bundle of interventions care pathway embedded in a newly modelled integrated transitional care HF program was both feasible and successful in a broad-spectrum cohort of real-world patients with chronic HF with a recent HF admission. The clinical pathway designed to address health care needs of high-risk patients fit to attend outpatient day-case hospital-based visits was associated with a significant reduction of the composite endpoint of all-cause death and all-cause hospitalization in the short and mid-term, as it decreased the primary composite outcome of all-cause hospitalization or all-cause mortality at 30, 90 and 180 days after a discharge for HF in this subset of patients. This outcome was maintained in subgroups of patients at higher risk of adverse outcomes, such as the elderly, those with CKD or categorized as CCP. These results of this work are relevant since confirm the feasibility and potential benefits of transitional care programs beyond the 30-day barrier and should encourage policy makers to promote the implementation of such integrated care HF programs for patients in the most vulnerable phase: the post-discharge period.

HF is a chronic syndrome with a highly negative impact on mortality, morbidity, and health-related quality of life [1,3]. It constitutes a leading cause of hospital readmissions in developed countries, especially in the first few months after an index hospitalization for HF, with 30- and 180 days rehospitalization rates reaching 20–30% and 50% respectively [25,26]. Readmissions represent a social and sanitary burden, as they are reportedly associated to poorer outcomes and high economical costs [27,28]. Thus, reduction of those rates in HF patients is a main goal of all Health Care Systems worldwide. Among readmission prevention strategies, hospital-based HFU along with integrated care, play a key role by offering a multidisciplinary structured follow-up that offers an early post discharge visit, telemonitoring, patient education and empowerment, evidence-based treatment uptitration and open access to day-care hospital enabling ambulatory intravenous treatments [11]. Consequently, the latest guidelines for the treatment of HF from the European Society of Cardiology rate these organizational models as a class I recommendation with level A evidence and recommend its generalized implementation [22].

HF care models inspired by the chronic care model are called disease management programs for HF, also known as heart failure programs or heart failure units. These units aim to ensure that the scientific evidence is applied homogeneously without variation, thereby helping to raise the bar for quality of care and increase the equity of our health care system [11]. However, the success of a HF program depends on the type of measures included in it, because it is not a specific and non-modifiable intervention such as a pharmacological treatment, but rather a package of measures that obtain the maximum performance if applied correctly, coordinately, and promptly in the most vulnerable phase of heart failure, the early period after decompensation.

The basis of the new HF care model presented in this work is the evolution of a comprehensive transitional care model developed and successfully implemented by our group in a different health care setting [21]. Our transitional care model is based on 4 pillars: 1) a systematic in-hospital intervention that includes a holistic assessment to confirm diagnosis, clinical stabilization and coordinate an adequate discharge planning; 2) early visit post-discharge to confirm euvolemia and review medication; 3) nurse-led structured follow-up based on clinical pathways that ensure evidence-based interventions and promote patient education and empowerment; 4) planning of advanced to primary care, advanced HF units or end-of-life care as needed. Throughout this process, the HF specialist nurse plays a key role by coordinating the various health levels to ensure continuity of care, promoting patient self-care, titrating prognostic medication, and monitoring treatment adherence, as well as the occurrence of decompensations or adverse effects [29,30]. In the current work we evaluate the effectiveness of the evolution of this transitional model adding new elements to improve clinical outcomes in these patients. First, includes universal detection of patients to adequately promote an effective discharge planning in all patients admitted for HF with a dedicated autonomous physician-nurse team that screen all hospital admissions in a daily basis and support the in-hospital care teams in HF evaluation, management, comprehensive psycho-social evaluation, and continuity of care of these patients. Second, implements a double check transition of care process with primary care: electronical communication of care plan (single check) and face-to-face weekly meetings. In these meetings, hospital-based HF nurses coordinate with primary care case-manager teams composed by nurses and physicians, to refine a shared care plan for each patient (case management) and close the loop back on previously discharged patients. Third, all post discharge settings (outpatient day-case hospital, home-based or primary care center) deliver care on the basis of dedicated, structured clinical pathways adapted to the needs of patients and coordinated by nurses. Our current study evaluates the potential effect of one such pathways: the one dedicated to high-risk patients followed in the outpatient hospital-based facilities of our comprehensive HF program. In this evaluation we confirm the benefits of the 7 key steps to be carried out by the HF nurse tin this care setting to ensure improved outcomes throughout the patient’s journey (**S1 Table**).

The results of our study validate the theoretical model of transitional care in HF above described, confirming that the application of the program is feasible and may improve clinical outcomes in different health care areas. The repercussion of implementing a nurse-led multidisciplinary HF program is visible in the short term and it lasts over time. Based on these findings, it is necessary to promote the widespread deployment of multidisciplinary HF programs and to encourage their continuous evaluation in each specific environment.

### Limitations

This study has some limitations that need to be commented. First, this is a single health care area natural experiment, which makes external validation of the results difficult. However, the study is based on the application of a model previously developed and implemented in different healthcare setting where it also showed successful outcomes. Therefore, we consider the benefits of the model implemented to be reasonably proven. Second, all the confounding biases typical of a natural experiment need to be taken into consideration. Third, due to the retrospective nature of the study, neither the treatment of the patients at the time of inclusion, nor the therapeutic changes that occurred during follow-up could be evaluated. Thus, we were unable to assess how treatment differed between cohorts and how this may have influenced the results. HFrEF patients complete a 6-month intervention to allow treatment up-titration and the follow-up period can be extended if the prespecified program goals are not attained. However, a high proportion (43.9%) of patients had a LVEF ≥50%, a population in which no treatment had shown a decrease in readmissions or mortality at the time when the study was carried out. In these patients a significant decrease in the main outcome was also observed and therefore we consider that the effect of the inclusion in a HF program is maintained regardless of LVEF or the possibility of optimizing neurohormonal treatment. And finally, in this study we only analysed the effects of the specific clinical pathway designed for high-risk patients attending the outpatient HF clinic based in our HF day-case hospital. High risk patients needing home-based care and intermediate-to-low risk patients needing primary care-based follow-up were not included in the current analyses. However, these patients have received primary-care based, nurse-led interventions with dedicated clinical pathways that will be evaluated in further studies.

## Conclusions

A new 7-step nurse-led bundle of interventions care model embedded in a comprehensive primary-care integrated HF program improves the composite endpoint of all-cause death and all-cause hospitalization beyond the 30-day period and demonstrates benefits in the mid and long-term in the post-discharge period, also in high-risk populations.

The reduction of events was mainly driven by a sustained decrease in CV and HF hospitalization. Benefits in all-cause mortality were also observed when the full model including a more exhaustive discharge planning process was implemented.

## Supporting information

S1 Fig Study evaluation periods. (DOCX)

S2 Fig Adjusted time-to-event estimates for the primary endpoint according to treatment group in special subgroups of patients. (DOCX)

S3 Fig Event-free survival cumulative curves for the primary endpoint (all-cause death or all-cause re-hospitalization) according to the discharge planning modality compared to usual care. (DOCX)

S4 Fig Event-free survival cumulative curves for the primary endpoint (all-cause death or all-cause re-hospitalization) according to the discharge planning modality. (DOCX)

S1 Table Nurse-led day-care hospital based 7-step bundle of transitional interventions. (DOCX)

S2 Table Data sources, coding criteria for the study, and data quality control. (DOCX)

S3 Table Multivariable adjusted Cox regression analyses evaluating the outcome of HF program management vs. usual care on clinical outcomes in special subgroups of patients. (DOCX)

S4 Table Proportion of events and multivariable adjusted logistic regressions analyses evaluating the outcome of the modality of discharge planning (single check vs. double check) in patients referred to the nurse-led hospital-based HF programme on primary and secondary efficacy endpoints at 6 months after inclusion. (DOCX)

## Abbreviations

HF: Heart Failure
MAHFE: Major Adverse Heart Failure Events
ICD: International Classification of Diseases
AMG: Adjusted Morbidity Group
HFU: Heart Failure Unit
LVEF: Left Ventricular Ejection Fraction
CPGs: Clinical Practice Guidelines
CV: Cardio Vascular
CKD: Chronic Kidney Disease
CCP: Complex Chronic Patients
eFGR: Estimated Glomerular Filtration Rate
NKF: National Kidney Foundation
NYHA: New York Heart Failure
BMI: Body Mass Index
HFpEF: Heart Failure with Preserved Ejection Fraction
HFrEF: Heart Failure with Reduced Ejection Fraction
Hb: Haemoglobin

## References

[1] Farré N, Vela E, Clèries M, Bustins M, et al. Real world heart failure epidemiology and outcome: A population-based analysis of 88,195 patients. *PLoS One*. 2017 Feb 24;12(2):e0172745. doi: 10.1371/journal.pone.0172745 28235067 PMC5325273

[2] Roger VL. Epidemiology of heart failure. Circ Res. 2013 Aug 30;113(6):646–59. doi: 10.1161/CIRCRESAHA.113.300268 23989710 PMC3806290

[3] Garay A, Tapia J, Anguita M, Formiga F, et al. Gender Differences in Health-Related Quality of Life in Patients with Systolic Heart Failure: Results of the VIDA Multicenter Study. J Clin Med. 2020 Aug 31;9(9):2825. doi: 10.3390/jcm9092825 32878281 PMC7563299

[4] Groenewegen A, Rutten FH, Mosterd A, et al. Epidemiology of heart failure. Eur J Heart Fail. 2020 Aug;22(8):1342–1356. doi: 10.1002/ejhf.1858 32483830 PMC7540043

[5] Cainzos-Achirica M, Capdevila C, Vela E, et al. Individual income, mortality and healthcare resource use in patients with chronic heart failure living in a universal healthcare system: A population-based study in Catalonia, Spain. *Int J Cardiol* 2019; 277: 250–257. doi: 10.1016/j.ijcard.2018.10.099 30413306

[6] Farré N, Vela E, Clèries M, et al. Medical resource use and expenditure in patients with chronic heart failure: a population-based analysis of 88 195 patients. *Eur J Heart Fail* 2016; 18: 1132–1140. doi: 10.1002/ejhf.549 27108481

[7] Lofman I, Szummer K, Dahlstrom U, et al. Associations with and prognostic impact of chronic kidney disease in heart failure with preserved, mid-range, and reduced ejection fraction. Eur J Heart Fail. 2017;19:1606–1614. doi: 10.1002/ejhf.821 28371075

[8] Dharmarajan K, Rich MW. Epidemiology, Pathophysiology, and Prognosis of Heart Failure in Older Adults. Heart Fail Clin. 2017 Jul;13(3):417–426. doi: 10.1016/j.hfc.2017.02.001 28602363

[9] Gheorghiade M, Vaduganathan M, Fonarow GC, et al. Rehospitalization for heart failure: problems and perspectives. J Am Coll Cardiol. 2013;61(4):391–403. doi: 10.1016/j.jacc.2012.09.038 23219302

[10] Calero-Molina E, Hidalgo E, Rosenfeld L, et al. The relationship between self-care, long term-mortality and heart failure hospitalization: insights from a real-world cohort study Eur J Cardiovasc Nurs 2021 Feb doi: 101093/eurjcn/zvab011 [Ahead of print]. 10.1093/eurjcn/zvab011 34008849

[11] Comín-Colet J, Enjuanes C, Lupón J, et al. Transitions of Care Between Acute and Chronic Heart Failure: Critical Steps in the Design of a Multidisciplinary Care Model for the Prevention of Rehospitalization. *Rev Española Cardiol (English Ed*) 2016; 69: 951–961. doi: 10.1016/j.rec.2016.05.001 27282437

[12] Feltner C, Jones CD, Cené CW, et al. Transitional care interventions to prevent readmissions for persons with heart failure: a systematic review and meta-analysis. Ann Intern Med. 2014 Jun 3;160(11):774–84. doi: 10.7326/M14-0083 24862840

[13] Pacho C, Domingo M, Núñez R, et al. Early Postdischarge STOP-HF-Clinic Reduces 30-day Readmissions in Old and Frail Patients With Heart Failure. Rev Esp Cardiol (Engl Ed). 2017 Aug;70(8):631–638. English, Spanish. doi: 10.1016/j.rec.2017.01.003 28215922

[14] Jaarsma T, van der Wal MH, Lesman-Leegte I, et al. Coordinating Study Evaluating Outcomes of Advising and Counseling in Heart Failure (COACH) Investigators. Effect of moderate or intensive disease management program on outcome in patients with heart failure: Coordinating Study Evaluating Outcomes of Advising and Counseling in Heart Failure (COACH). Arch Intern Med. 2008 Feb 11;168(3):316–24. doi: 10.1001/archinternmed.2007.83 18268174

[15] Gorthi J, Hunter C, Mooss A, et al. Reducing heart failure hospital readmissions: a systematic review of disease management programs. Cardiol Res 2014 Jan;5(5):126–138. doi: 10.14740/cr362w 28348710 PMC5358117

[16] Takeda A, Martin N, Taylor RS, et al. Disease management interventions for heart failure. Cochrane Database Syst Rev. 2019 Jan 8;1(1):CD002752. doi: 10.1002/14651858.CD002752.pub4 30620776 PMC6492456

[17] Qiu X, Lan C, Li J, et al. The effect of nurse-led interventions on re-admission and mortality for congestive heart failure: A meta-analysis. Medicine (Baltimore). 2021 Feb 19;100(7):e24599. doi: 10.1097/MD.0000000000024599 33607793 PMC7899814

[18] Ojeda S, Anguita M, Delgado M, et al. Short- and long-term results of a program for the prevention of readmissions and mortality in patients with heart failure: are effects maintained after stopping the program? Eur J Heart Fail. 2005 Aug;7(5):921–6. doi: 10.1016/j.ejheart.2005.05.009 16051519

[19] Monterde D, Vela E, Clèries M, et al. Los grupos de morbilidad ajustados: nuevo agrupador de morbilidad poblacional de utilidad en el ámbito de la atención primaria [Adjusted morbidity groups: A new multiple morbidity measurement of use in Primary Care]. Aten Primaria. 2016 Dec;48(10):674–682. Spanish. doi: 10.1016/j.aprim.2016.06.003 27495004 PMC6879171

[20] Servei Català de la Salut. Pla de Salut de Catalunya 2011–2015. Barcelona: Generalitat de Catalunya; 2012.

[21] Comín-Colet J, Verdú-Rotellar JM, Vela E, et al. Efficacy of an integrated hospital-primary care program for heart failure: a population-based analysis of 56,742 patients. Rev Esp Cardiol (Engl Ed). 2014 Apr;67(4):283–93. doi: 10.1016/j.rec.2013.12.005 24774591

[22] McDonagh TA, Metra M, Adamo M, et al. 2021 ESC Guidelines for the diagnosis and treatment of acute and chronic heart failure. Eur Heart J. 2021 Sep 21;42(36):3599–3726. doi: 10.1093/eurheartj/ehab368 34447992

[23] Chronic Condition Indicator (CCI) for ICD-10-CM. https://www.hcup-us.ahrq.gov/toolssoftware/chronic_icd10/chronic_icd10.jsp.

[24] Catalonia Health Department. TERMCAT, Centre de terminologia. *Terminologia de la cronicitat* [en línia]. Barcelona: TERMCAT, Centre de Terminologia, cop. 2013. Avaliable from: http://www.termcat.cat/ca/Diccionaris_En_Linia/160/.

[25] Krumholz HM, Lin Z, Keenan PS, et al. Relationship between hospital readmission and mortality rates for patients hospitalized with acute myocardial infarction, heart failure, or pneumonia. JAMA. 2013 Feb 13;309(6):587–93. doi: 10.1001/jama.2013.333 23403683 PMC3621028

[26] Dharmarajan K, Hsieh AF, Lin Z, et al. Diagnoses and timing of 30-day readmissions after hospitalization for heart failure, acute myocardial infarction, or pneumonia. JAMA. 2013 Jan 23;309(4):355–63. doi: 10.1001/jama.2012.216476 23340637 PMC3688083

[27] Bundkirchen A., Schwinger R.H.G. Epidemiology and economic burden of chronic heart failure. *Eur*. *Heart J*. *Suppl*. 2004;6:D57–D60. doi: 10.1016/j.ehjsup.2004.05.015

[28] Fernandez-Gasso L, Hernando-Arizaleta L, Palomar-Rodríguez JA, Abellán-Pérez MV, Pascual-Figal DA. Trends, causes and timing of 30-day readmissions after hospitalization for heart failure: 11-year population-based analysis with linked data. Int J Cardiol. 2017 Dec 1;248:246–251. doi: 10.1016/j.ijcard.2017.07.094 28801153

[29] Riley JP, Astin F, Crespo-Leiro MG, Deaton CM, et al. Heart Failure Association of the European Society of Cardiology heart failure nurse curriculum. Eur J Heart Fail. 2016 Jul;18(7):736–43. doi: 10.1002/ejhf.568 27220672

[30] Oyanguren J, Garcia-Garrido L, Nebot-Margalef M, et al. Steering Committee on behalf of the ETIFIC research team group. Noninferiority of heart failure nurse titration versus heart failure cardiologist titration. ETIFIC multicenter randomized trial. Rev Esp Cardiol (Engl Ed). 2020 Jun 24:S1885–5857(20)30186-9. doi: 10.1016/j.rec.2020.04.016 32591295

