# Correction: Breaking the 30-day barrier: Long-term effectiveness of a nurse-led 7-step transitional intervention program in heart failure

**DOI:** 10.1371/journal.pone.0309956

**Published:** 2024-08-30

**Authors:** Lidia Alcoberro, Pedro Moliner, Joan Vime, Santiago Jiménez-Marrero, Alberto Garay, Sergi Yun, Alexandra Pons-Riverola, Raúl Ramos-Polo, Mar Ras-Jiménez, Marta Tajes, Encarna Hidalgo, Esther Calero, Marta Ruiz, Nuria José-Bazán, Carles Ferre, Cristina Delso, Laia Alcober, Cristina Enjuanes, Josep Comin-Colet

The published paper is missing the following Acknowledgments section: The authors would like to acknowledge the CERCA Program/Generalitat de Catalunya for their institutional support.
