# Supplementary figures and images for: Breaking the 30-day barrier: Long-term effectiveness of a nurse-led 7-step transitional intervention program in heart failure
Source: PLoS One. 2023 Feb 7;18(2):e0279815. doi: 10.1371/journal.pone.0279815 (PMC9904494; doi:10.1371/journal.pone.0279815)

**Supplementary Figure 1. Study evaluation periods**


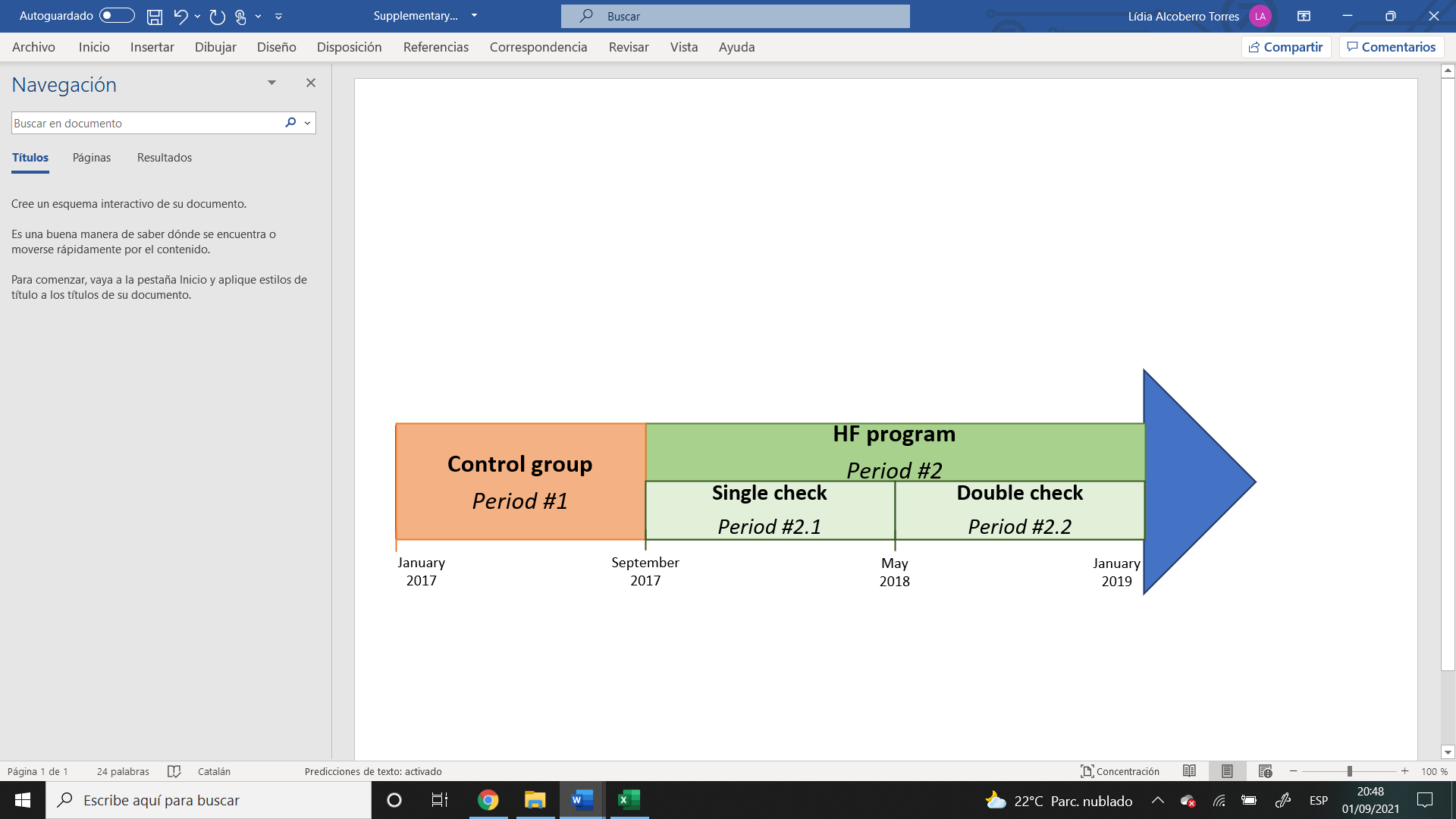

Supplement: S1 Fig — (DOCX) [file pone.0279815.s001.docx]
