# Supplementary material for: Breaking the 30-day barrier: Long-term effectiveness of a nurse-led 7-step transitional intervention program in heart failure
Source: PLoS One. 2023 Feb 7;18(2):e0279815. doi: 10.1371/journal.pone.0279815 (PMC9904494; doi:10.1371/journal.pone.0279815)
Supplement: S1 Table — (DOCX) [file pone.0279815.s005.docx]

**Supplementary Table 1. Nurse-led day-care hospital based 7-step bundle of transitional interventions.**

| Step 1 | Discharge planning: active in-hospital search of hospitalised patients, universal detection, integral evaluation. |
| --- | --- |
| Step 2 | Double check approach for the coordination of care plan with primary care teams:   - Single check: 48 h pre-discharge electronic communication of care plan to the primary care team. - Double check: face to face joint weekly sessions with primary care case managers liaising with primary care teams. |
| Step 3 | Early (48h) post-discharge contact (telephone). |
| Step 4 | Early nurse-led post-discharge visit. |
| Step 5 | 6 month-telephone nurse-based structured follow-up: Telemonitoring, uptitration, empowerment. |
| Step 6 | Open access (hot line) clinic in the day-care hospital enabling IV diuretics treatment in ambulatory patients. |
