# Supplementary material for: Breaking the 30-day barrier: Long-term effectiveness of a nurse-led 7-step transitional intervention program in heart failure
Source: PLoS One. 2023 Feb 7;18(2):e0279815. doi: 10.1371/journal.pone.0279815 (PMC9904494; doi:10.1371/journal.pone.0279815)
Supplement: S2 Table — (DOCX) [file pone.0279815.s006.docx]

**Supplementary Table 2. Data Sources, Coding Criteria for the Study, and Data Quality Control**

| **Data sources** | |
| --- | --- |
| *Hospital admissions and readmissions* | Registry of the Minimum Data Set corresponding to Acute Care Hospitals for 2017-2019. This population-based registry collects information on all the discharges recorded in the hospitals of Catalonia, focusing on administrative and clinical care data (length of stay, diagnoses, and procedures). |
| *Mortality* | Mortality data, including the date of death, were obtained from the Mortality Registry of Catalonia, provided by the Health Department of the Government of Catalonia. |
| **Diagnosis coding (International Classification of Diseases – 10-CM)** | |
| *Hospital admission for heart failure* |  |
| 398.91 | Rheumatic heart failure (congestive); left-sided |
| 402.x1 | Hypertensive heart disease with heart failure |
| 404.x1 | Hypertensive heart disease and hypertensive nephropathy with congestive heart failure |
| 404.x3 | Hypertensive heart disease and hypertensive nephropathy with congestive heart failure and chronic kidney disease |
| 428.0 | Congestive heart failure, unspecified; right side is secondary to left side |
| 428.1 | Left heart failure; acute pulmonary edema |
| 428.2x | Systolic heart failure |
| 428.3x | Diastolic heart failure |
| 428.4x | Combined systolic and diastolic heart failure |
| *Quality control of the data sources* |  |
| Registry of the Minimum Data Set of Acute Care Hospitals | The registry has an automatic data validation system. An external audit is carried out periodically to ensure the quality and veracity of the data |
| Diagnostic coding | The hospitals have trained encoders to ensure professional performance of the coding process. The encoders hold biannual consensus meetings on coding regulations, coinciding with the updates of the International Classification of Diseases. |
