# Supplementary material for: Breaking the 30-day barrier: Long-term effectiveness of a nurse-led 7-step transitional intervention program in heart failure
Source: PLoS One. 2023 Feb 7;18(2):e0279815. doi: 10.1371/journal.pone.0279815 (PMC9904494; doi:10.1371/journal.pone.0279815)
Supplement: S3 Table — (DOCX) [file pone.0279815.s007.docx]

**Supplementary Table 3. Multivariate adjusted Cox regression analyses evaluating the impact of HF program management vs. usual care on clinical outcomes in special subgroups of patients.**

|  | **Patients ≥80 years old** | | **Chronic complex patients** | | **Chronic Kidney Disease** | |
| --- | --- | --- | --- | --- | --- | --- |
| **Primary Endpoint** | *Hazard Ratio (95% CI) | p-value | *Hazard Ratio (95% CI) | p-value | *Hazard Ratio (95% CI) | p-value |
| All-cause death or all-cause hospitalization | 0.42 (0.23-0.77) | 0.005 | 0.41 (0.26-0.66) | <0.001 | 0.32 (0.21-0.49) | <0.001 |
| **Secondary Endpoints** |  |  |  |  |  |  |
| HF hospitalization | 0.19 (0.09-0.40) | <0.001 | 0.29 (0.17-0.50) | <0.001 | 0.19 (0.11-0.33) | <0.001 |
| CV hospitalization | 0.22 (0.11-0.44) | <0.001 | 0.32 (0.19-0.53) | <0.001 | 0.22 (0.14-0.36) | <0.001 |
| All-cause hospitalization | 0.42 (0.23-0.77) | 0.005 | 0.40 (0.25-0.64) | <0.001 | 0.32 (0.21-0.49) | <0.001 |
| All-cause death |  | 0.612 | 0.36 (0.02-7.82) | 0.519 | 0,22 (0.02-2.03) | 0.989 |
| All-cause death or CV hospitalization | 0.23 (0.12-0.46) | <0.001 | 0.33 (0.20-0.55) | <0.001 | 0.23 (0.14-0.36) | <0.001 |
| All cause death of HF hospitalization | 0.24 (0.12-0.50) | <0.001 | 0.30 (0.18-0.52) | <0.001 | 0.19 (0.11-0.31) | <0.001 |
