# Supplementary material for: Breaking the 30-day barrier: Long-term effectiveness of a nurse-led 7-step transitional intervention program in heart failure
Source: PLoS One. 2023 Feb 7;18(2):e0279815. doi: 10.1371/journal.pone.0279815 (PMC9904494; doi:10.1371/journal.pone.0279815)
Supplement: S4 Table — (DOCX) [file pone.0279815.s008.docx]

**Supplementary Table 4. Proportion of events and multivariate adjusted logistic regressions analyses evaluating the impact of the modality of discharge planning (single check vs. double check) in patients referred to the nurse-led hospital-based HF programme on primary and secondary efficacy endpoints at 6 months after inclusion.**

|  | **Unadjusted Analyses** | | | **Adjusted Analyses** | |
| --- | --- | --- | --- | --- | --- |
|  | Single check discharge planning | Double check discharge planning | p-value | *Odds Ratio (95% CI) | p-value |
| **Primary Endpoint 180 days** | | | | | |
| All-cause death or all-cause hospitalization | 50 (27%) | 22 (16%) | 0,021 | 0.53 (0.32-0.86) | 0,011 |
| **Secondary Endpoints 180 days** | | | | | |
| All-cause hospitalization | 51 (28%) | 23 (17) | 0,023 | 0.57 (0.32-1.01) | 0,056 |
| All-cause death or CV hospitalization | 33 (18%) | 13 (10%) | 0,037 | 0.48 (0.24-0.94) | 0,035 |
| All cause death of HF hospitalization | 28 (15%) | 11 (8%) | 0,058 | 0.48 (0.23-1.01) | 0,054 |

*Comparison of double check discharge planning vs. single check discharge planning (reference category).
